# Effect of human papillomavirus education on human papillomavirus knowledge and gynecologic cancer awareness among women aged 18–49: a randomized controlled study

**DOI:** 10.1590/1806-9282.20260313

**Published:** 2026-08-07

**Authors:** Büşra Demirdağ, Reyhan Metin Ayhan, Merve Nur Keleş, Hümeyra Öğeç, Melike Nur Ulugedik, Mustafa Yasin Duran

**Affiliations:** 1 Karatay University, Faculty of Health Sciences – Konya, Türkiye.; 2 Karatay University License Graduate – Konya, Türkiye.; 3 Konya City Hospital, Department of Emergency Medicine – Konya, Türkiye.

**Keywords:** HPV, Health belief model, Health education, Human papillomavirus infections, Women’s health, Awareness

## Abstract

**OBJECTIVE:**

Human papillomavirus is a major cause of cervical and other human papillomavirus-related cancers, and education is essential for prevention. The aim of this study was to evaluate the effect of a structured human papillomavirus education program on women’s human papillomavirus knowledge, health beliefs, and gynecologic cancer awareness.

**METHODS:**

A randomized controlled study was conducted with 68 women assigned to intervention and control groups. The intervention included four weekly educational sessions, and data were collected before and after the program using validated scales.

**RESULTS:**

While baseline scores were comparable, post-test results showed significantly higher human papillomavirus knowledge, health beliefs (severity, benefits, and susceptibility), and gynecologic cancer awareness in the intervention group, with no change in perceived barriers.

**CONCLUSION:**

A brief, theory-based human papillomavirus education program effectively improves women’s human papillomavirus-related knowledge and awareness and may support community-based prevention efforts.

## INTRODUCTION

As the most prevalent sexually transmitted illness in the world, human papillomavirus (HPV) is a serious and expanding global public health concern. Most sexually active individuals are exposed to the virus at some point in their lives^1^. Among more than 150 HPV genotypes, 13 are classified as high-risk due to their carcinogenic potential^2^. Persistent HPV infection is responsible for more than 90% of cervical and anal cancers and a substantial proportion of oropharyngeal, penile, vaginal, and vulvar cancers^1^. Globally, approximately 570,000 women and 60,000 men are diagnosed each year with HPV-related cancers, with the highest burden observed in low- and middle-income countries^3^.

Cervical cancer remains an important public health problem in Turkey, where HPV infection is the primary etiological factor. Each year, more than 2,000 women are newly diagnosed with cervical cancer, and studies report a wide prevalence range for high-risk HPV types, particularly types 16 and 18^4^. The broad spectrum of cancers associated with HPV highlights that the infection is not only an individual health issue but also a major societal concern.

HPV-related diseases are largely preventable through vaccination and education. The World Health Organization aims to eliminate cervical cancer by achieving a 90% HPV vaccination rate among adolescent girls by 2030; however, global vaccination coverage remains low^5,6^. Factors such as limited knowledge, vaccine hesitancy, socioeconomic barriers, and misconceptions about vaccine safety contribute to low vaccination rates^7^. In Turkey, although HPV vaccines are licensed, they are not included in the national immunization program, further limiting access and uptake^8^.

For primary (vaccination) and secondary (screening) prevention strategies to be effective, individuals must have adequate knowledge and awareness of HPV^9^. However, studies consistently show insufficient HPV knowledge even among high-risk and educated populations^10^. Due to their increased risk of HPV exposure and their status as major recipients of preventative measures like vaccination and routine screening, women of reproductive age are more vulnerable to this disparity. Although educational interventions have been shown to significantly improve HPV-related knowledge and attitudes, the sustainability of this effect remains a challenge, emphasizing the need for structured and repeated education programs. There are few experimental research assessing the effects of structured HPV education programs that especially target women in Turkey between the ages of 18 and 49, despite the availability of effective preventive strategies. This is a significant gap in the literature, especially when it comes to producing data to inform national preventative initiatives.

Despite the preventable nature of HPV infection and related cancers, lack of awareness and misinformation continue to pose significant barriers. To enhance both individual and public health outcomes in this situation, well-thought-out educational programs catered to women in the reproductive age group are crucial. Therefore, this study aims to evaluate the effect of HPV education on HPV knowledge and gynecological cancer awareness among women aged 18–49. The findings are expected to contribute to the evidence supporting education-based interventions and to inform public health strategies in Turkey, where experimental studies targeting this age group remain limited.

### Research hypotheses

H1–1: HPV Infection Knowledge Scale scores of the intervention group will be higher than those of the control group.

H2–1: Health Belief Model Scale scores of the intervention group will be higher than those of the control group.

H2–3: Gynecologic cancer awareness levels of the intervention group will be higher than those of the control group.

## METHODS

### Type of the study

This study employed a pre-test–post-test design and was conducted as an experimental, randomized, and controlled trial. The study was carried out in accordance with the CONSORT 2017 standards.

### Study setting and sample

The research was conducted at a Women’s Community Center operating under a municipality in Konya. The center provides social, cultural, and educational activities aimed at supporting women’s health and well-being and operates between 09:00 and 17:00 on weekdays and weekends.

The study population consisted of married women of reproductive age registered at the community center. Sample size was calculated using the G*Power 3.1.9.2 program, resulting in a required sample of 68 participants (34 intervention, 34 control), assuming an effect size of 0.80, 90% power, and a 5% margin of error. Inclusion criteria were being literate and aged 18 years or older. Women who had previously participated in a similar program were excluded, and participants who failed to attend at least two educational sessions were removed from the study.

### Randomization

Participants were randomly assigned to intervention and control groups using a simple randomization method. Group allocation (A=intervention, B=control) was performed using an online random number generator. Randomization was completed immediately prior to data collection. An independent researcher created the randomization sequence, and following baseline evaluation, participants were divided into groups. Because of the nature of the intervention, blinding was not possible; however, both groups underwent standardized processes to reduce bias.

### Data collection tools

Data were collected using the Personal Information Form, the Human Papillomavirus Infection Knowledge Scale, the Health Belief Model Scale for Human Papillomavirus Infection and Vaccination (HPV-HBMS), and the Gynecological Cancers Awareness Scale (GCAS).

The Personal Information Form included questions on sociodemographic characteristics such as age, education level, marital status, income level, and parental education.

Human Papillomavirus Infection Knowledge Scale: The scale was originally developed by Kim^11^, with validity and reliability established internationally and later adapted into Turkish by Guvenc et al. The Turkish short form consists of 10 items with “true,” “false,” and “not sure” response options. Each correct answer is scored as one point, while incorrect or “not sure” responses receive zero points. Total scores range from 0 to 22, with higher scores indicating greater HPV knowledge. Cronbach’s alpha was reported as 0.88 in the original study and 0.85 in the Turkish adaptation^12^.

HPV-HBMS: The Human Papillomavirus Infection Knowledge Scale, originally developed by Kim, consists of true/false/I am not sure items scored as one for correct answers and zero for incorrect or unsure responses^11^. Higher scores indicate greater HPV knowledge. The scale demonstrated acceptable reliability in both the original and Turkish validation studies. The HPV-HBMS evaluates perceived severity, susceptibility, benefits, and barriers related to HPV infection and vaccination using a four-point Likert scale. Each subdimension is scored separately, with higher scores reflecting stronger health beliefs^12^.

GCAS: The GCAS assesses women’s awareness of gynecological cancers across four subdimensions. Higher total scores indicate greater awareness^13^.

### Variables

The independent variable of the study was HPV education. Dependent variables included HPV knowledge, health beliefs related to HPV infection and vaccination, and gynecological cancer awareness.

### Intervention

The HPV and cancer awareness education program was developed based on relevant literature and expert opinions from the Departments of Midwifery and Public Health Nursing. Using a planned instruction process and standardized presentation materials, the same trained researcher conducted each session to guarantee intervention uniformity. The program consisted of four weekly sessions, each lasting approximately 45 min. To encourage interaction, the seminars were held in person in small groups. Every session adhered to a set format that comprised an introduction, a topic presentation, a discussion, and a summary. The sessions addressed HPV transmission and risk factors, vaccination and prevention strategies, gynecological cancers and screening methods, and the importance of early diagnosis and healthy lifestyle behaviors. Educational methods included presentations, group discussions, and visual materials. A checklist was utilized to make sure that all of the scheduled subjects were addressed, and the same instructional materials (slides, visual aids, and key messages) were used in every session to improve consistency.

### Data collection procedure

Pre-test data were collected from both groups before the intervention. The intervention group received the educational program, while the control group received no intervention. Post-tests were administered to both groups 6 weeks after the pre-test. To reduce interruptions and guarantee anonymity, all information was gathered in a calm, private space.

### Data analysis

Data analysis was performed using IBM Statistical Package for the Social Sciences (IBM SPSS Statistics), version 21.0. Descriptive statistics were used to summarize participant characteristics and scale scores. Normality was assessed using the Kolmogorov-Smirnov test, skewness, and kurtosis values. Parametric or non-parametric tests were applied accordingly. Statistical significance was set at p<0.05.

### Ethical considerations

The study was conducted in accordance with the principles of the Declaration of Helsinki and complied with the ethical standards and regulations of Turkey. Ethical approval was obtained from a local Non-Interventional Clinical Research Ethics Committee Written informed consent was obtained from all participants prior to data collection. Participants were informed about the purpose of the study, the voluntary nature of participation, and their right to withdraw at any stage without any consequences. Confidentiality and anonymity were ensured throughout the study. After completion of the study, participants in the control group were offered access to a reinforcement educational program.

## RESULTS

Table 1 shows the comparison of HPV Infection Knowledge Scale scores between groups across measurement times. There was no significant difference between the groups at the pre-test (p>0.05). However, post-test scores were significantly higher in the intervention group compared to the control group (p<0.05). Within-group analysis revealed a significant increase in the intervention group (p<0.05), while no significant change was observed in the control group (p>0.05).

**Table 1:** Comparison of human papillomavirus Infection Knowledge Scale measurements at follow-up times by group.

	Group		Test statistics		
	Experimental	Control	Test value^‡^	p	η^2^
	n=34	n=34			
HPV Infection Information Scale					
Pre-test	4.73±2.77	4.29±2.36	0.705	0.483	0.082
Post-test	8.17±1.11	4.02±2.66	8.363	**0.000**	0.682
Test value^†^	**t=-7.765; p=0.000**	t=0.941; p=0.355			
Difference^&^ (final-initial)	2.03±1.58	0.14±0.81	12.280	**0.000**	0.369

^‡^Independent Samples t-test (t);

†Paired Samples t-test,

^&^Comparison of first and last score differences between groups. Summary statistics are presented as mean±standard deviation and median (minimum, maximum) for numerical variables, and as frequency (percentage) for categorical variables. Sections highlighted in bold indicate statistical significance (p<0.05). HPV: human papillomavirus.

The pre-test and post-test mean scores of the HPV-HBMS subscales by group are presented in Table 2. In the post-test, significant between-group differences were observed in the perceived severity, perceived benefits, and perceived susceptibility subscales (p<0.05). Within-group comparisons showed that post-test scores in these subscales were significantly higher than pre-test scores in the intervention group (p<0.05), whereas no significant differences were found in the control group. No significant between-group or with-in-group differences were observed in the perceived barriers subscale (p>0.05).

**Table 2:** Comparison of Health Belief Model Scale for Human Papillomavirus Infection and Vaccination measurements at follow-up times by group.

HPV-HBMS	Group		Test statistics		
	Experimental	Control	Test value^‡^	p	η^2^
	n=34	n=34			
Perception of seriousness					
Pre-test	11.64±3.06	11.38±2.87	0.368	0.714	0.002
Post-test	15.32±1.27	11.26±2.95	7.351	**0.000**	0.450
Test value^†^	t=6.806; **p=0.000**	t=0.412; p=0.683			
Difference^&^ (pre-post)	3.67±3.67	-0.11±1.66	6.209	**0.000**	0.369
Perception of barriers					
Pre-test	11.11±3.28	9.44±2.28	2.444	0.17	0.083
Post-test	10.35±1.98	9.73±2.39	1.159	0.250	0.020
Test value^†^	t=1.125; p=0.269	t=-1.068; p=0.293			
Difference^&^ (pre-post)	-0.76±3.96	0.29±1.60	-1.444	0.153	0.031
Perceived benefit					
Pre-test	7.44±2.24	7.94±1.93	-0.983	0.329	0.014
Post-test	10.85±1.43	8.05±1.98	6.649	**0.000**	0.401
Test value^†^	t=-7.084; **p=0.000**	t=-0.453; p=0.653			
Difference^&^ (pre-post)	3.41±2.80	0.11±1.51	6.022	**0.000**	0.355
Perception of sensitivity					
Pre-test	4.50±1.39	5.00±5.08	6.251	**0.000**	0.033
Post-test	7.11±1.03	5.00±1.37	-1.489	0.141	0.372
Test value^†^	t:7.937 p=0.000	t:0.533 p=0.598			
Difference^&^ (pre-post)	2.61±1.92	0.08±0.96	6.855	**0.000**	0.416

^‡^Independent samples t-test (t);

^†^paired samples t-test;

^&^comparison of first and last score differences between groups. Summary statistics are presented as mean±standard deviation and median (minimum, maximum) for numerical variables, and as frequency (percentage) for categorical variables. Sections highlighted in bold indicate statistical significance (p<0.05). HPV-HBMS: Health Belief Model Scale for Human Papillomavirus Infection and Vaccination.

Table 3 presents the comparison of GCAS scores by group. While pre-test scores did not differ significantly between groups (p>0.05), post-test scores were significantly higher in the intervention group than in the control group (p<0.05). Within-group analysis demonstrated a significant increase in GCAS scores in the intervention group (p<0.05), whereas no significant change was observed in the control group. Additionally, post-test–pre-test difference scores were significantly higher in the intervention group compared to the control group (p<0.05).

**Table 3:** Comparison of Gynecological Cancer Awareness Scale measurements at follow-up times according to groups.

	Group		Test Statistics		
	Experimental	Control	Test value^‡^	p	η^2^
	n=34	n=34			
JİKFÖ					
Pre-test	151.64±14.49	153.97±17.67	-0.593	0.555	0.005
Post-test	184.23±10.62	156.17±21.30	**6.872**	**0.000**	**0.417**
Test value^†^	**t=-10.769; p=0.000**	t=-0.950; p=0.3549			
Difference^&^ (pre-post)	32.58±17.64	2.20±13.53	**7.967**	**0.000**	**0.490**

^‡^Independent samples t-test (t);

^†^paired samples t-test;

^&^Comparison of first and last score differences between groups. Summary statistics are presented as mean ± standard deviation and median (minimum, maximum) for numerical variables, and as frequency (percentage) for categorical variables. Sections highlighted in bold indicate statistical significance (p<0.05).

## DISCUSSION

HPV education is an effective primary prevention strategy that enhances knowledge of transmission, prevention, and vaccination, thereby reducing HPV-related cancer risk. This study showed that HPV education significantly improved women’s HPV-related knowledge, health beliefs, and gynecological cancer awareness.

In the present study, HPV Infection Knowledge Scale scores of the intervention group increased significantly after the education and were higher than those of the control group. These findings are consistent with previous studies showing that educational interventions significantly improve knowledge related to HPV infection, cervical cancer, Pap smear testing, and vaccination, while cross-sectional studies among adolescents reporting low baseline knowledge levels regarding HPV and sexually transmitted infections further emphasize the need for such educational interventions^14,15,16,17^. Similarly, education supported by visual and video-based materials has been reported to enhance awareness of cervical cancer screening and HPV vaccination^18^. Web-based and classroom-based educational programs have also been shown to effectively increase HPV awareness^19,20^. Overall, these findings highlight the effectiveness of structured educational programs in improving women’s knowledge and awareness of HPV and cervical cancer.

The current study also found significant improvements in health beliefs related to HPV following the intervention. Posttest scores of the intervention group were significantly higher in the perceived severity, perceived benefits, and perceived susceptibility subscales, whereas no significant change was observed in perceived barriers. Similar findings have been reported in previous studies, which demonstrated increased intentions toward HPV vaccination after educational interventions^21^. Differences in vaccination intention levels across countries and populations suggest that cultural and socioeconomic factors play an important role in shaping attitudes toward HPV vaccination^11^. These results indicate that planned educational programs can positively influence health beliefs and vaccination intentions.

In addition, the Gynecological Cancers Awareness Scale scores of the intervention group increased significantly after the education and were higher than those of the control group. This finding is supported by studies reporting that educational interventions improve awareness of gynecological cancer symptoms and risk factors^18^. However, it contrasts with the findings of Ampofo et al., who reported no significant change in knowledge levels following education^22^. Overall, the results suggest that well-structured educational interventions are an effective approach to increasing women’s awareness and understanding of gynecological cancers.

## CONCLUSION

This randomized controlled study demonstrates that a structured HPV education program significantly improves women’s HPV knowledge, health beliefs, and gynecologic cancer awareness. Short-term, multi-session educational interventions implemented in community-based settings may support primary and secondary prevention strategies for HPV-related cancers. Future studies should include larger and more diverse samples, longer follow-up periods, and behavioral outcomes such as vaccination uptake and screening participation.

## Data Availability

The datasets generated and/or analyzed during the current study are available from the corresponding author upon reasonable request.
